# Use of standardized outcome measures among physical therapists in the United States: A cross-sectional survey study

**DOI:** 10.1371/journal.pone.0330528

**Published:** 2025-08-20

**Authors:** Anat Kristal, Ignacio A. Gaunaurd, Sara J. Morgan, Geoffrey S. Balkman, Rachael E. Rosen, Rana Salem, Alyssa M. Bamer, Brian J. Hafner

**Affiliations:** 1 Department of Physical Therapy, University of Miami Miller School of Medicine, Florida, United States of America; 2 Department of Rehabilitation Medicine, University of Washington School of Medicine, Washington, United States of America; 3 Research Department, Gillette Children’s Specialty Healthcare, Minnesota, United States of America; Iran University of Medical Sciences, IRAN, ISLAMIC REPUBLIC OF

## Abstract

As value-based care becomes increasingly central to healthcare delivery in the United States, physical therapists (PTs) are expected to use outcome measures (OMs) to demonstrate meaningful improvements in patient health. Although OMs play a critical role in supporting value-based practice, prior research suggests they are not routinely used, and limited attention has been given to how resource availability may influence their implementation, particularly for performance-based tests. This study aimed to assess PTs’ use of OMs, including their perceptions of value, motivations for use, and the resources available for administration. An online survey was used to evaluate outcomes measurement practices of PTs in the United States. Invitations were mailed to a random sample of American Physical Therapy Association members. Frequencies and percentages were calculated. Nearly all respondents (n = 514) used OMs with their patients, but performance-based tests were used more than self-report surveys (97% and 83%, respectively). Most respondents agreed that OMs were within their scope of practice (>95%) and could be administered with knowledge they possessed (>88%). However, less than 50% agreed OMs are administered in a standardized manner across the profession. Most respondents (89%) indicated that performance-based tests “help me make clinical decisions,” but fewer (61%) said the same about self-report surveys. Respondents were also willing to dedicate more time to performance-based tests than self-report surveys (up to 20 and 10 minutes, respectively). Most respondents (>80%) indicated they had smaller rooms and short hallways (≤10m) for OM administration. Results of this study indicate PTs in the United States have the knowledge, time, and space to administer many of the OMs recommended for use in physical therapy. While PTs use both types of OMs, performance-based tests are often perceived to have greater clinical value. Obtaining consensus on specific protocols for administering OMs may help to address concerns about the lack of standardization across the profession.

## Introduction

There is an increasing shift in the United States towards value-based care that emphasizes improving outcomes that matter most to patients. Value-based care is viewed as a common goal among patients, clinicians, employers, insurers, and government organizations [1]. Value, in this context, can be established by measuring improvements in patients’ health relative to the expenses incurred to attain those outcomes [2]. Outcome measures (OMs) are standardized tools that physical therapists (PTs) and other health professionals can use to evaluate aspects of patients’ health before, during, and after an intervention [3]. Use of OMs allows PTs to engage in value-based practices by objectively assessing tangible changes in a patient’s health outcomes over the course of their care [4].

Despite the benefits of using OMs in physical therapy, prior evidence has indicated that they are not administered routinely in clinical practice. In 2009, Jette and colleagues surveyed a large national sample of PTs in the United States and found that fewer than 50% of the respondents used standardized OMs with their patients [5]. A key limitation of this study, however, is that the survey developed by the investigators asked PTs only about their use and opinions of one type of OM, health status questionnaires (i.e., self-report surveys). The survey administered did not include questions about performance-based tests, another type of OM commonly used in physical therapy practice. The findings reported by Jette *et al*., therefore, likely underestimate the overall use of standardized OMs among PTs in the United States in the late 2000s. This notion is supported by evidence from a study of OM use among PTs in Korea published in late 2023 [6]. Lim and colleagues surveyed 220 PTs and found that just over half (n = 126, 57.3%) of respondents used self-report surveys alone or in combination with performance-based tests in their practices. However, a larger majority (n = 165, 75.0%) used performance-based tests either alone or in combination with self-report surveys.

Including performance-based tests in assessments of OM utilization among PTs is important because performance-based tests provide information that is distinct from and complementary to self-report surveys [7,8]. However, performance-based tests also require additional resources like access to space (e.g., open rooms or long corridors) and equipment (e.g., chairs or stairs) and may, therefore, be more challenging to administer in some of the settings in which PTs practice. In 2022, Morgan and colleagues conducted a study to evaluate the use of both self-report surveys and performance-based tests among prosthetists in the United States [9]. While respondents (n = 375) indicated relatively high utilization of standardized OMs overall (93%), limited time and space were reported to be barriers to routine use of OMs. As the developed survey included questions specific to the time, space, and equipment available to prosthetists for outcomes measurement, the authors were able to propose specific OMs suited to these constraints. Understanding the resources available to PTs can similarly aid in identifying OMs suitable for administration in the clinical environments in which PTs practice.

The goal of this study was, therefore, to assess the current state of outcomes measurement in the PT profession, including professional expectations, practice habits, and general opinions regarding the value of self-report surveys and performance-based tests. Additionally, PTs were queried regarding the availability of resources, such as time, space, and equipment (including mobile technology) for administering both types of OMs in clinical practice.

## Materials and methods

A cross-sectional study was conducted between March 2023 and April 2023 with PTs across the United States. A local institutional review board reviewed the study protocol and determined it qualified for exempt status.

### Participants

The target population for this study was PTs who provide care regularly to adult and/or pediatric patients in the United States. Contact information for a random sample of 4,000 PTs from select American Physical Therapy Association (APTA) special interest groups (i.e., acute care, federal PT, geriatrics, oncology, orthopedics, pediatrics, private practice, and sports physical therapy) was obtained to identify potential participants.

### Sample size justification

A total sample of 380 survey respondents was needed to obtain a reliable estimate of the practice habits and opinions of the targeted sample. The sample size target was calculated based on the total number of PTs in the selected special interest groups (N = 29,736), a 95% confidence level, and a 5% margin of error [10].

### Survey

The original survey [9] developed by Morgan et al. was rigorously developed through a literature review and formal interviews [11] with physiatrists, physical therapists, and prosthetists in the U.S., then pilot tested with prosthetists to assess clarity, relevance, and usability. For the current study, the survey was adapted by the research team, two of whom are physical therapists with expertise in outcome measure development, to ensure relevance to the physical therapy profession. Adaptations were minor and focused primarily on customizing demographic items such as work environment and education.

Questions in the survey addressed a variety of barriers and facilitators to outcomes measurement that are relevant to PTs, including opinions about OMs, reasons for using OMs, and the time, space, and equipment available for administering OMs. The survey also included questions about respondents’ practice settings, patient load, clinical training, and personal characteristics. Modifications were made to the original survey to make the response options relevant to PTs (e.g., the practice setting categories were changed to reflect those used by the APTA [12]). The revised survey used for the present study is included in the Supplementary Material (S1 Appendix).

### Study procedures

The survey was developed and administered using a Research Electronic Data Capture (REDCap) [13,14] application hosted at the University of Washington. PTs from the provided APTA mailing list were sent invitations to participate in the study along with a link to an online questionnaire. A follow-up letter was sent to non-respondents three weeks after the initial invitation. Eligibility was self-assessed via two screening questions regarding participants’ age (i.e., at least 18 years or older) and practice status (i.e., currently licensed and practicing as a physical therapist). To ensure eligibility, respondent names were crosschecked with the APTA mailing list and relevant states’ licensing authorities. Survey responses were reviewed, and participants were contacted by email or phone to obtain missing data.

### Analysis

Descriptive statistics were used to characterize the study sample. Frequencies and percentages were presented for categorical data. To facilitate comparisons with U.S. prosthetists [8], the frequency of OMs use categories was collapsed (i.e., “often” and “always” were grouped as “routine” use; “rarely” and “sometimes” were grouped as “occasional” use). To assess the sample’s representativeness with the PT profession, participants’ characteristics (i.e., age, gender, race, and ethnicity) were tabulated and compared with the most recent APTA Practice Profile Analysis sample [10]. Continuous variables, such as age, time in practice, and times of appointments, were assessed for normality using IBM SPSS Statistics v26 (Armonk, NY). Data were tabulated with SPSS and graphed with Microsoft Excel 2021 (Redmond, WA).

## Results

### Participants

Invitations were sent to 3674 individuals from the APTA mailing list with valid addresses. 595 respondents completed the self-screening process. 71 respondents did not complete the survey (15 were not eligible, 32 were eligible but did not start the survey, and 24 were eligible but did not complete the survey). No records were removed for inaccuracies or missing data. A total of 524 respondents completed the survey, with a response rate of 14.3%. 10 respondents indicated that they do not typically treat patients and were removed from the final dataset (n = 514) used for this analysis (Table 1).

**Table 1:** Demographic and professional characteristics of the study sample (N = 514).

Characteristic	n	%	Characteristic	n	%
**Gender**			**Employer**		
Man	164	32	Acute care hospital	76	15
Woman	339	66	Hospital-based outpatient facility or clinic	144	28
Non-binary	1	<1	Private outpatient office or group practice	179	35
More than one gender	1	<1	Skilled nursing facility (SNF)/long-term care	13	3
Not reported	9	2	Patient’s home/home care	32	6
**Ethnicity**			Inpatient rehabilitation facility (IRF)	13	3
Hispanic or Latino	20	4	School system (pre-secondary)	22	4
Not Hispanic or Latino	473	92	Academic institution (post-secondary)	16	3
Not reported	21	4	Health and wellness facility	6	1
**Race**			Research center	0	0
American Indian or Alaskan Native	0	0	Industry	1	<1
Asian	26	5	Other	12	2
Black or African American	5	1	**Practice location**		
Native Hawaiian/Pacific Islander	0	0	Midwest	115	22
White	442	86	Pacific	81	16
More than one race	10	2	Southeast	118	23
Prefer to self-describe	4	1	Northeast	102	20
Not reported	27	5	Southwest	43	8
**Highest education level**			Rocky Mountains	47	9
Bachelor’s degree (BPT)	40	8	Noncontiguous U.S.	8	2
Master’s degree (MSPT)	67	13	**Typical patient load**		
Clinical doctorate (DPT)	385	75	Do not treat patients on a weekly basis	15	3
PhD or other advanced degree	22	4	1-10 patients per week	59	11
**Age**			11-20 patients per week	83	16
24-34 years	163	32	21-30 patients per week	108	21
35-44 years	137	27	31-40 patients per week	125	24
45-54 years	98	19	More than 40 patients per week	124	24
55-64 years	88	17	**Patient characterization**		
65 years or older	19	4	See adults primarily	411	80
Not reported	9	2	See children primarily	84	16
**Time in physical therapy practice**			Even distribution of children and adults	19	4
Up to 5 years	122	24			
6-10 years	91	18			
11-20 years	115	22			
21-30 years	102	20			
31 years or more	84	16			

The median age of the sample was 41 years (range 25–73 years), and the median time in practice was 13 years (range <1–47 years). The majority of respondents were women (66%) and of White race (86%). The study sample was similar to that reported in the APTA Practice Profile Analysis based on demographic characteristics such as median age (study sample: 41 years, practice analysis sample: 43 years), woman gender or female sex (66%, 65%), race (American Indian or Alaskan Native: 0%, < 1%; Asian: 5%, 7%; Black or African-American: 1%, 3%; Native Hawaiian or Other Pacific Islander: 0%, < 1%; White, 86%, 84%) and ethnicity (Hispanic or Latino: 4%, 4%) [12].

Most survey respondents worked in outpatient settings (63%); fewer worked in acute care (15%), long-term care (11%), or other (11%) settings. The majority of respondents (80%) treated primarily adult patients, and many (68%) had a patient load of more than 20 patients per week. Nearly all respondents (95%) reported they had training in outcomes measurement. Most (65%) indicated they had received formal training while at college/university, but more than half of respondents also said they had taken continuing education courses (53%), received informal training from others (52%), or self-trained (57%) in the administration of OMs.

### Use of outcome measures

The large majority (93%) of respondents indicated that use of one or both types of OMs (i.e., performance-based tests or self-report surveys) was expected or encouraged at their clinic/facility. Moreover, nearly all (97%) respondents indicated that they used OMs with their patients. While most respondents (83%) used self-report surveys, almost all (97%) used performance-based tests. Among those respondents who used performance-based tests, the large majority (96%) reported they were directly responsible for administering the tests. The others relied on another clinician or staff member to administer the test. Among respondents whose practices used self-report surveys, fewer (only 61%) indicated they were responsible for administering the surveys. Front office staff members were also sometimes responsible (35%) for survey administration. Very few respondents (<4%) relied upon other clinicians, assistants, or volunteers to administer performance-based tests or self-report surveys to their patients.

### Motivations for using outcome measures

**Fig 1:**
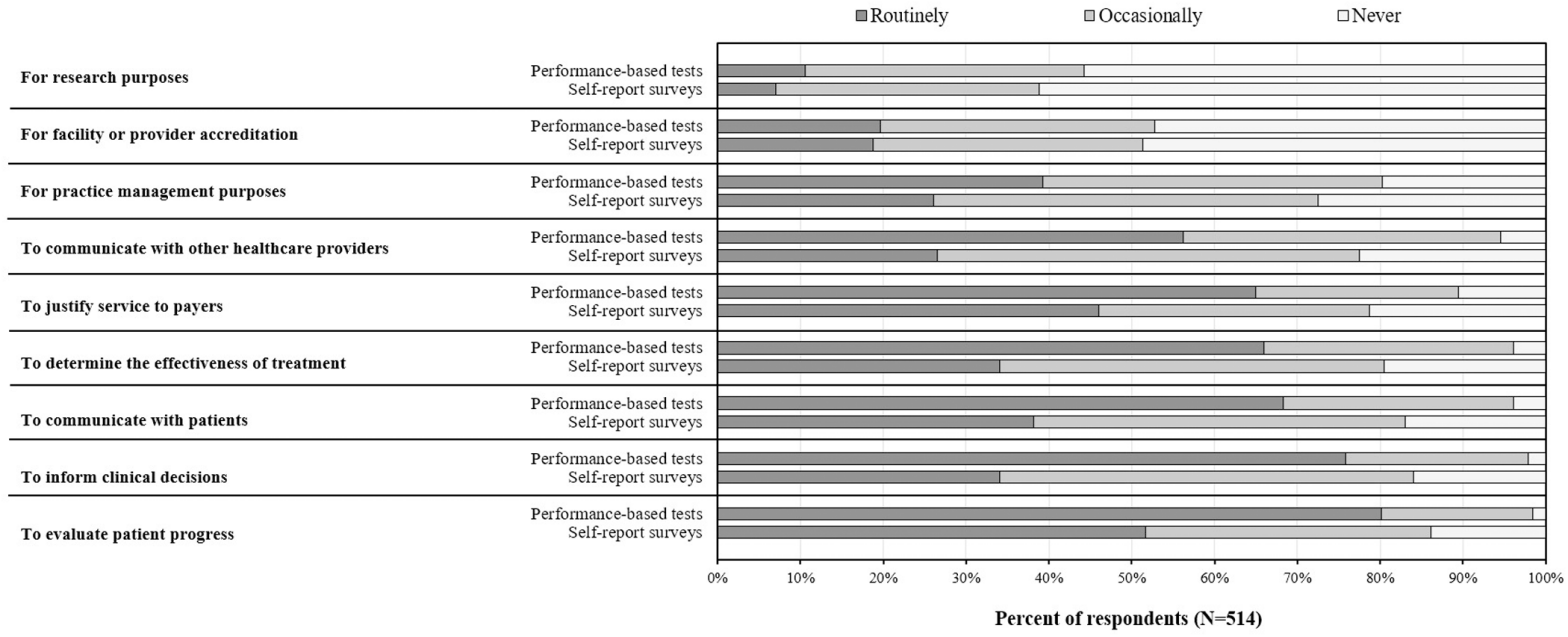
Reasons for using outcome measures.

The three most endorsed reasons for the routine use of performance-based tests (Fig 1) included “to evaluate patient progress” (80%), “to inform clinical decisions” (76%), and “to communicate with patients” (68%). The three most endorsed reasons for the routine use of self-report surveys were “to evaluate patient progress” (52%), “to justify service to payers” (46%), and “to communicate with patients” (38%) (Fig 1). The two least endorsed reasons for routine use of OMs were similar for performance-based tests and self-report surveys “for facility or provider accreditation” (20% and 19%, respectively) and “for research purposes” (11% and 7%, respectively).

### Perspectives on use of outcome measures

The large majority of respondents agreed or strongly agreed that performance-based tests and self-report surveys “are within my scope of practice” (98% and 95%, respectively), “can be administered with knowledge that I have” (96% and 88%), and “are inexpensive” (82% and 85%) (Table 2). However, less than half the sample agreed or strongly agreed that these types of OMs “are administered in a standardized way throughout my profession” (48% and 46%, respectively). Perspectives on the different types of OMs differed markedly in three key areas, however. Most respondents agreed or strongly agreed that performance-based tests are “a good use of my time” (90%), “help me make clinical decisions” (89%), and “are meaningful or motivating to my patients” (78%). Agreement was lower for self-report surveys, with 52%, 61%, and 47% endorsing these statements, respectively.

**Table 2:** Perspectives on use of outcome measures.

Statements regarding the use of outcome measures	Percent* of respondents (N = 514) who agreed with statements on performance-based tests			Percent* of respondents (N = 514) who agreed with statements on self-report surveys		
	Agree or strongly agree	Neutral	Disagree or strongly disagree	Agree or strongly agree	Neutral	Disagree or strongly disagree
Are within my scope of practice	98	2	<1	95	5	<1
Can be administered using knowledge that I have	96	4	1	88	10	2
Help me make clinical decisions	89	9	2	61	29	10
Are a good use of my time	90	8	2	52	33	15
Are a good use of my patients’ time	84	14	2	53	35	11
Can be administered by me without additional help	83	15	2	N/A	N/A	N/A
Are inexpensive	82	12	6	85	13	2
Can be administered with equipment available in my clinic	83	13	4	N/A	N/A	N/A
Are easy to make a part of my routine	82	14	4	63	22	15
Are easy for me to conduct	78	18	4	N/A	N/A	N/A
Are meaningful or motivating to my patients	78	19	3	47	40	13
Can be administered with space available in my clinic	76	16	8	N/A	N/A	N/A
Help me communicate with other health care providers	75	21	4	47	36	16
Are easy to put into my medical record system	73	14	13	65	18	17
Are easily scored and interpreted	72	22	6	69	23	8
Are helpful in acquiring insurance approvals	65	29	6	42	46	12
Can be quickly administered	52	29	19	60	29	11
Are administered in a standardized way throughout my profession	48	28	23	46	30	24

*Percentages may not sum to 100% due to rounding. N/A = statements deemed not applicable for self-report surveys.

### Time available for administering outcomes measures

Respondents indicated their median (25^th^-75^th^ percentiles) clinical appointment times for evaluation, re-evaluation, and discharge were as follows: 50 min (45–60), 40 min (30–45), and 30 min (30–45), respectively. Respondents were generally willing to spend more time administering performance-based tests than self-report surveys during these clinical appointments (Table 3). Only about one-quarter of the respondents (24–27%) indicated they were willing to spend 20 minutes or more administering performance-based tests during each of these clinical appointments, but many more (62–64%) were willing to spend between 5 and 20 minutes with these tests. Less than one-tenth of respondents (6–9%) were willing to spend 10 minutes or more administering self-report surveys; however, the large majority of respondents (85–87%) were willing to spend less than 10 minutes administering surveys at these appointments. Finally, nearly all respondents (96–98%) said they would be willing to spend time communicating results from a performance-based test or self-report survey to a patient and documenting results in the patient’s record, but most (83–86%) indicated they would not spend more than 10 minutes doing either of these activities.

**Table 3:** Time available to administer, communicate, and document outcome measures.

Clinical activity	Type of appointment	Percent* of respondents (N = 514) who would allocate the noted time for each clinical activity					
		None	< 5 min	5-10 min	10-20 min	20-30 min	> 30 min
**Administering performance-based tests**	Initial evaluation	1	8	29	34	15	12
	Re-evaluation	2	11	34	29	16	9
	Discharge	4	11	34	27	14	9
**Administrating self-report surveys**	Initial evaluation	6	53	33	5	3	1
	Re-evaluation	7	61	26	3	2	1
	Discharge	8	60	26	4	2	1
**Communicating outcome measure results to a patient**	Initial evaluation	3	51	35	10	2	<1
	Re-evaluation	3	55	31	9	1	<1
	Discharge	4	52	32	10	2	1
**Documenting outcome measure results in a patient’s clinical record**	Initial evaluation	2	61	23	9	2	2
	Re-evaluation	2	64	22	9	2	1
	Discharge	4	64	22	7	3	1

*Percentages may not sum to 100% due to rounding.

### Clinical resources available for administering outcome measures

The large majority of respondents (81%) reported having access to spaces where patients could walk short distances (i.e., up to 10m) (Table 4). Fewer respondents indicated they had indoor spaces where patients could walk longer distances (e.g., 30m) or run/jog (<38% and 46%, respectively). However, 69% of respondents indicated they had access to a treadmill. More than half of respondents (50–92%) indicated they had access to small-to-medium sized rooms or narrow hallways where they could administer performance-based tests. Fewer (7–42%) had access to wide hallways, L-shaped areas, or gyms where they could administer these tests. Most respondents (84%) also had access to some type of stairs, but far fewer (38%) had access to a low-grade ramp (with or without handrails). Two-thirds of respondents (66%) noted they had access to a tablet computer for administering OMs.

**Table 4:** Space available to administer outcome measures.

Space and equipment	Percent of respondents (N = 514) with space or equipment available
**Spaces for specific activities**	
Space to walk 5m and turn around	92
Space to walk 10m and turn around	81
Space to walk 30m and turn around	38
Space to run or jog	46
Outdoor space to walk over uneven surfaces	58
Gym/large indoor space	50
Large outdoor space	36
**Spaces with specific dimensions**	
3.5m x 1.5m room	92
3.5m x 3.5m room	65
8m x 3m space	54
9m x 1.5m hallway	71
12m x 1.5m hallway	50
16-m x 3-m wide hallway	26
9-m + 5-m “L” shaped	42
14-m x 14-m gym	7
**Stairs and ramps**	
Set of therapy stairs with 3–5 steps	61
Staircase with 6–8 steps	52
Staircase with 9 or more steps	52
Low-grade (up to 5 degrees) ramp without handrail	28
Low-grade (up to 5 degrees) ramp with handrail	22

## Discussion

The present study examined outcomes measurement practices among PTs in the United States. Almost all respondents (97%) reported using standardized OMs (i.e., performance-based tests and/or self-report surveys) with their patients. These findings confirm our expectation that Jette and colleagues likely underestimated the overall use of OMs among PTs, as their survey focused solely on self-report surveys and did not include questions about respondents’ use of performance-based tests [5]. Results from the present study also suggest that PTs now use self-report surveys more frequently than they did 15 years ago (i.e., more than 80% use now compared to less than 50% previously) [5]. The marked increase in the use of self-report surveys is likely driven by changes in physical therapy education standards, particularly the requirement to select and competently administer a variety of OMs during examination, evaluation, and diagnosis [15]. This educational requirement aligns with the ongoing shift toward value-based care in the U.S. healthcare system, which aims to improve the quality of patient care by rewarding positive patient outcomes [16].

PTs’ reasons for using OMs appear to be largely clinically motivated, as respondents’ most frequently endorsed reasons for using self-report surveys and performance-based tests were evaluating patient progress, informing clinical decisions, and communicating with patients. Many respondents also indicated they used both types of OMs to justify service to providers. Collectively, these findings reinforce the role that routine measurement of patient outcomes can play in value-based care [4]. Overall, few respondents described using OMs for other reasons like practice management, facility accreditation, or research. This implies that outcomes measurement may be more effective at the patient level than at the practice or profession levels. However, it should be noted that the use of self-report surveys for reasons related to practice management and research in the present study was nearly twice that reported by PTs in 2009 [5], suggesting an evolving appreciation of OMs for these practices- or profession-level activities.

Although PTs in the present study used both types of OMs with their patients, there did appear to be a notable preference for performance-based tests. In addition to a modest difference in overall utilization (i.e., 97% vs. 83%), respondents indicated they used performance-based tests more often than self-report surveys for all of the clinical activities included in the survey (Fig 1). PTs in South Korea also reported greater use of performance-based tests than self-report surveys (75.0% and 57.3%, respectively) [6], albeit with lower overall utilization than PTs in the present study practicing in the United States. Less frequent use of self-report surveys may reflect PT’s perception that these OMs offer limited value towards patient management. When compared to performance-based tests, fewer respondents in the present study viewed self-report surveys as a good use of their time (90% vs. 52%), helpful for making decisions (89% vs. 61%), or meaningful to their patients (78% vs. 47%). Similar findings reported in the literature suggest that the perceived limited value of self-report surveys remains a key barrier to their use [17]. As few respondents in the present study were willing to dedicate more than five minutes to administering self-report surveys (32%−41%) compared to performance-based tests (86–90%), these concerns appear valid. Given that many respondents in the present study perceived self-report surveys as less clinically useful than performance-based tests, efforts should be made to demonstrate their value, both individually and in combination with performance-based tests. For example, self-report measures can provide rich information about how an individual functions in their home and/or community [7,8]. Such information can then be compared to results from performance-based tests, which provide greater information about a patient’s functional capacity in a controlled environment. Collectively, these distinct but complementary pieces of information can provide a more comprehensive view of a patient’s overall functioning. Education on the purpose and benefits of self-report surveys may be useful but may not be sufficient on its own to promote widespread changes in practice habits among clinicians [18]. More comprehensive approaches (e.g., audit and feedback from management [19]) may be required to stimulate the routine use of self-report surveys in physical therapy practice.

While the large majority of respondents (95–98%) in this study reported that administration of performance-based tests and self-report surveys was within their scope of practice and could be done using knowledge they possessed, far fewer (46–48%) agreed that these measures are administered in a standardized manner across the profession. These findings are consistent with opinions recently reported by U.S. prosthetists [9], raising additional concerns about OM standardization across different healthcare professions. Evidence to substantiate concerns with the standardization of performance-based tests exists. A study of PTs working with patients with neurological conditions found wide variability in how the 6-minute walk test (6MWT) was administered, including configuration and length of the walkway, as well as how distance was measured (i.e., following the patient with a measuring wheel or counting laps of a fixed distance) [20]. Similarly, a study of prosthetists working with patients with limb loss found notable differences in walkway length, presence of potential obstacles, how distance was measured, and instructions provided to patients during administration of the 2-minute walk test (2MWT) [21]. While evidence to support respondents’ concerns of standardization with self-report surveys is limited, issues common to all OMs (e.g., time of day they are administered, patients’ exposure to recent experiences, variations in scoring methods) can still affect survey responses and scores. Given that PTs and prosthetists identified concerns with both types of OMs, future efforts should focus on obtaining cross-disciplinary consensus on standardized protocols for administering both performance-based tests and self-report surveys to patients that might be seen by clinicians from multiple disciplines.

Examination of the spaces available to PTs for outcomes measurement was also an objective of the present study. The large majority of respondents (>80%) reported access to rooms or spaces up to 10m in length, spaces sufficient for administering OMs advocated for use in physical therapy practice [22]. For example, a small room or 5-meter space can accommodate performance-based tests like the Timed-Up and Go (TUG) test [23] or the Berg Balance Scale (BBS) [24], OMs that have been used to assess basic mobility, balance, and fall risk in a range of clinical populations [25,26]. Ten-meter spaces are generally sufficient to administer tests like the 10-meter walk test (10mWT) [27], a performance-based test designed to measure walking speed – a health indicator that is widely recognized for its broad clinical utility [28]. Access to these moderately-sized spaces also allows PTs to administer components of comprehensive performance-based tests, like the Functional Gait Assessment (FGA) [29], that require patients to walk short distances under different conditions (e.g., while stepping over a small obstacle or while walking with a narrow base-of-support). The FGA is often recommended for use in PT practice, given its broad applicability to a variety of clinical populations [30]. Far fewer (≤50%) respondents in the present study had access to larger spaces, like long hallways, gyms, or spaces to run or jog. While performance-based tests rarely necessitate larger spaces, tests of walking capacity, like the 6-minute walk test (6MWT) [31], require a straight 30-meter hallway according to published protocols [32]. PTs without access to these larger spaces may be required to modify the test [20], contributing to the issues of non-standardization raised by many respondents in the present study.

Resources like stairs may also be needed to administer select OMs. Stairs are required to administer performance-based tests that include stair tasks like the FGA [29] or standalone tests of stair performance like the Timed Stair Test (TST) [33] or the Stair Climb Test (SCT) [34]. While the large majority of respondents (>80%) in the present study reported having access to stairs, only about half had access to the longer stairwells (i.e., 9 or more stairs) typically required for timed stair tests like the TST or SCT [33]. For PTs with access to only shorter sets of stairs, timed stair tests with as few as 4–6 steps are available and may be viable alternatives [35,36]. However, variability in the types and lengths of stairs available to PTs may be one reason why tests that allow flexibility in the number of stairs (e.g., the FGA [29]) are more often included among those OMs recommended for use in clinical practice [22].

Computerized administration of OMs has been advocated as a means to improve the overall ease and efficiency of outcomes measurement [5]. While only 7% of respondents in a prior study reported using computers for administering and analyzing OMs [5], data from the present study suggests that two-thirds of PTs have access to tablet computers with internet access for this purpose. This marked increase in the use of mobile devices may help promote administration of outcome measures on digital platforms that integrate with existing electronic medical records systems in rehabilitation settings [37]. Mobile devices and outcome measure applications have the potential to enhance the efficiency and reduce the administrative burden of outcomes measurement by standardizing instructions, automatically scoring OMs, generating reports, and providing the ability to directly transmit or download reports into patients’ electronic medical records. As the majority of respondents in the present study acknowledged they had limited time (<5 min) to communicate OM results to patients or document OM results in patients’ clinical records, use of mobile applications – particularly those that are directly integrated with electronic health record systems – may offer the means to more efficiently collect, convey, and document health outcomes in day-to-day practice. However, research is needed to determine whether mobile devices (i.e., tablets or smartphones) and applications can facilitate use of OMs in routine physical therapy practice.

Limitations to the present study include the targeted sample (i.e., APTA members from select special interest groups) and a relatively low (i.e., 14.3%) response rate, which may introduce a response bias, as those who chose to participate could differ systematically from non-respondents. For instance, participants may have had more exposure to or interest in the topic, which could have influenced their responses and, in turn, the study findings. Although the targeted sample size was exceeded, it is possible that the sample was biased toward PTs most interested in using OMs in clinical practice. If so, these results may overestimate the use of OMs among PTs in the U.S. However, the study included a large sample of PTs with characteristics (e.g., age, gender, race, and ethnicity) closely aligned with those reported for the profession in the APTA Practice Profile Analysis [12], suggesting that study respondents are generally representative of APTA members and PTs in the U.S. Future studies may consider strategies such as sending additional mail reminders and incentivizing respondents to complete the survey in order to improve the survey response rate. Another limitation is that most respondents worked in one of three settings (i.e., acute care hospitals, hospital-based outpatient facilities, or private outpatient offices/ group practices). Relatively few respondents worked in settings like skilled nursing or inpatient rehabilitation facilities. Thus, additional research is needed to determine if the results of this study generalize to PTs working in these settings. Lastly, this study did not investigate specific performance-based tests or self-report surveys used by PTs in routine clinical practice. Understanding which OMs are most frequently used or considered valuable for specific patient populations can enhance knowledge of current clinical practices, identify gaps in OM use, and inform strategies to optimize assessment and care across diverse patient groups. Future research should assess the type of OMs most commonly used by PTs for different patient populations, ideally identifying those with the greatest perceived value.

## Conclusion

Collectively, the results of this study offer a comprehensive view of the current state of outcomes measurement among physical therapists in the United States. Almost all respondents in the present study reported using OMs with their patients, suggesting outcomes measurement has become a standard of practice in physical therapy, shaped in part by professional expectations. While the use of self-report surveys has grown over the past 15 years, they remain underused relative to performance-based tests, reflecting prevailing practice habits in physical therapy. This study highlights the perception among clinicians that self-report surveys offer limited clinical value, which may hinder their use. Efforts to support implementation should include strategies that help demonstrate the relevance of these tools in clinical decision-making.

Despite efforts by the physical therapy profession to make recommendations on OMs suited to specific patients or settings, PTs still perceive a lack of standardization with respect to how self-report surveys and performance-based tests are administered. Work is needed to obtain consensus on the specific protocols for administering these different types of OMs, ideally in partnership with other professionals who provide rehabilitation care. Improving standardization may help promote more consistent use of outcome measures in practice and support broader implementation efforts. The large majority of PTs appear to have access to the spaces and resources needed to administer performance-based tests that are often recommended for use in daily practice. Many PTs also report access to mobile devices that could be used to streamline administration, scoring, and interpretation of OMs, but research is needed to confirm that these technologies can be successfully integrated into routine care.

## Supporting information

S1 Appendix Physical therapist outcomes measures survey. (PDF)

S2 File Dataset. (XLSX)
