# Supplementary material for: Use of standardized outcome measures among physical therapists in the United States: A cross-sectional survey study
Source: PLoS One. 2025 Aug 20;20(8):e0330528. doi: 10.1371/journal.pone.0330528 (PMC12367168; doi:10.1371/journal.pone.0330528)
Supplement: S1 Appendix — (PDF) [file pone.0330528.s001.pdf]

Appendix 1: Physical Therapist Outcomes Measures Survey

This survey will ask you questions about your clinical environment and use of outcome measures (i.e., **self-report surveys** and **performance-based tests**).

QUESTIONS ABOUT YOUR CLINICAL ENVIRONMENT

In which city and state do you work? (if you work at more than one location, please identify the location where you work most often)

City: ____________________

State: _____

How would you characterize your employer? (select one)

- ○ Acute care hospital
- ○ Hospital-based outpatient facility or clinic
- ○ Private outpatient office or group practice
- ○ Skilled nursing facility (SNF) / long-term care
- ○ Patient’s home / home care
- ○ School system (pre-school / primary / secondary)
- ○ Academic institution (post-secondary)
- ○ Health and wellness facility
- ○ Research center
- ○ Industry
- ○ Inpatient rehabilitation facility (IRF)
- ○ Other ________________________________

QUESTIONS ABOUT YOUR CLINICAL LOAD

How many individual patients, regardless of diagnosis, do you treat per week? (select one)

- ○ I do not treat patients on a weekly basis
- ○ 1-10 patients
- ○ 11-20 patients
- ○ 21-30 patients
- ○ 31-40 patients
- ○ More than 40 patients

How would you characterize the patients you typically treat? (select one)

- ○ I do not typically treat patients
- ○ I treat primarily children
- ○ I treat primarily adults
- ○ I treat an even distribution of children and adults

QUESTIONS ABOUT OUTCOMES MEASUREMENT AT YOUR CLINIC

At my clinic/facility, use of <u>self-report surveys</u> is:

- ○ An expectation
- ○ Encouraged
- ○ Not an expectation or encouraged

At my clinic/facility, use of <u>performance-based tests</u> is:

- ○ An expectation
- ○ Encouraged
- ○ Not expectation or encouraged

Who is <u>primarily</u> responsible for administering <u>self-report surveys</u> to your patients? (choose one)

- ○ Me
- ○ Another clinician or practitioner
- ○ Clinical assistants (e.g., PTAs)
- ○ Front office staff
- ○ Trainee (e.g., residents, students)
- ○ Other: ______________________________
- ○ We do not currently administer self-report surveys at my facility

Who is <u>primarily</u> responsible for administering <u>performance-based tests</u> to your patients? (choose one)

- ○ Me
- ○ Another clinician or practitioner
- ○ Clinical assistants (e.g., PTAs)
- ○ Front office staff
- ○ Trainee (e.g., residents, students)
- ○ Other: ______________________________
- ○ We do not currently administer performance-based tests at my facility

QUESTIONS ABOUT YOUR CLINICAL TIME

How much time do you spend in a <u>typical</u> initial, re-evaluation, and discharge clinical appointment? (if unsure, please make your best guess)

Initial evaluation ________ minutes

Re-evaluation ________ minutes

Discharge ________ minutes

Please indicate the *<u>maximum</u>* time you would be willing to spend on the following CLINICAL ACTIVITIES. (choose one per row)

	Type of appointment	None	Up to 5 min	5 - 10 min	10 - 20 min	20 - 30 min	More than 30 min
**Administering self-report surveys?**	Initial evaluation	○	○	○	○	○	○
	Re-evaluation	○	○	○	○	○	○
	Discharge	○	○	○	○	○	○
**Administering performance-based tests?**	Initial evaluation	○	○	○	○	○	○
	Re-evaluation	○	○	○	○	○	○
	Discharge	○	○	○	○	○	○
**Communicating outcome measure results to the patient?**	Initial evaluation	○	○	○	○	○	○
	Re-evaluation	○	○	○	○	○	○
	Discharge	○	○	○	○	○	○
**Documenting outcome measure results in the patient's clinical record?**	Initial evaluation	○	○	○	○	○	○
	Re-evaluation	○	○	○	○	○	○
	Discharge	○	○	○	○	○	○

QUESTIONS ABOUT YOUR SPACE AND EQUIPMENT

We are interested in how much space you have in your clinic to administer <u>performance-based tests</u>. Could you readily access and easily clear an <u>open space</u> of the following dimensions for this purpose? (choose one for each space)

Note: A standard chair with arms has been placed in each space for scale.

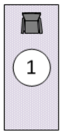<br>**3.5m x 1.5m**<br>(11.5ft x 4.9ft)	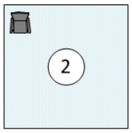<br>**3.5m x 3.5m**<br>(11.5ft x 11.5ft)	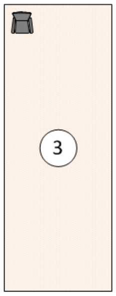<br>**8m x 3m**<br>(26.2ft x 9.8ft)	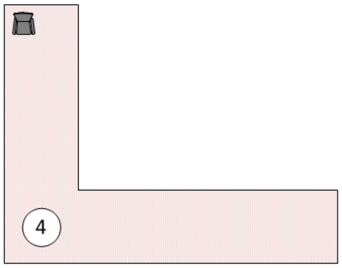<br>**9m x 2m + 5m x 2m**<br>(29.5ft x 6.6ft and 16.4ft x 6.6ft)
Space available?<br>☐ Yes ☐ No	Space available?<br>☐ Yes ☐ No	Space available?<br>☐ Yes ☐ No	Space available?<br>☐ Yes ☐ No

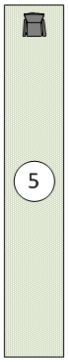<br>**9m x 1.5m**<br>(29.5ft x 4.9ft)	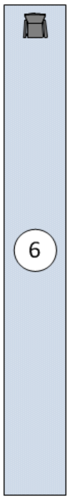<br>**12m x 1.5m**<br>(39.4ft x 4.9ft)	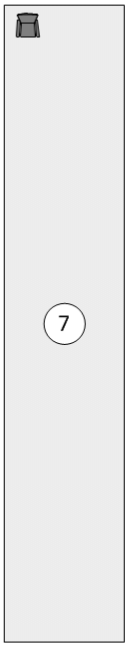<br>**16m x 3m**<br>(52.5ft x 9.8ft)	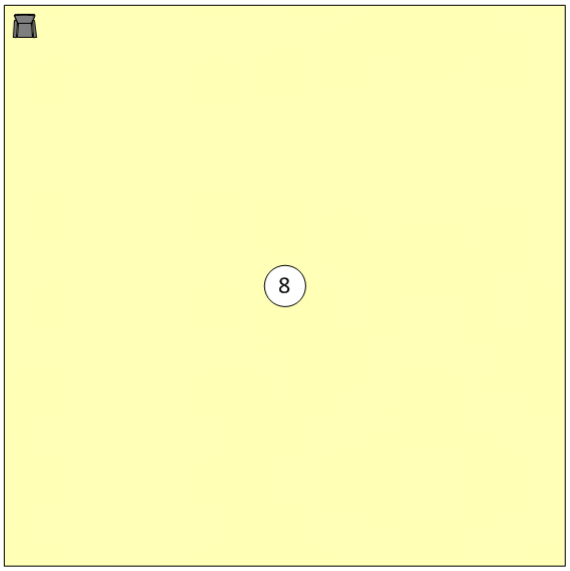<br>**14m x 14m**<br>(45.9ft x 45.9ft)
Space available?<br>☐ Yes ☐ No	Space available?<br>☐ Yes ☐ No	Space available?<br>☐ Yes ☐ No	Space available?<br>☐ Yes ☐ No

Please indicate whether you could readily access any of these to administer a performance-based test: (select all that apply)

- ☐ A gym/large indoor space
- ☐ A large outdoor space
- ☐ Space where a patient could walk 5m (16 feet) and turn around
- ☐ Space where a patient could walk 10m (33 feet) and turn around
- ☐ Space where a patient could walk 30m (98 feet) and turn around
- ☐ Space where a patient could run or jog
- ☐ Outdoor space where a patient could walk over uneven surfaces
- ☐ A set of therapy stairs with 3-5 steps
- ☐ A staircase with 6-8 steps
- ☐ A staircase with 9 or more steps
- ☐ A low-grade (up to 5 degrees) ramp without a handrail
- ☐ A low-grade (up to 5 degrees) ramp with a handrail
- ☐ A treadmill
- ☐ A tablet computer with wireless internet access

QUESTIONS ABOUT YOUR USE OF OUTCOME MEASURES

Please indicate HOW OFTEN you use self-report surveys for the following purposes.

Purpose:	Never	Rarely	Sometimes	Often	Always
To inform clinical decisions	○	○	○	○	○
To evaluate patient progress	○	○	○	○	○
To communicate with patients	○	○	○	○	○
To justify service to payers	○	○	○	○	○
To communicate with other healthcare providers	○	○	○	○	○
For research purposes	○	○	○	○	○
To determine the effectiveness of a treatment/intervention	○	○	○	○	○
For practice management purposes (e.g., quality improvement)	○	○	○	○	○
For facility or provider accreditation	○	○	○	○	○

Please indicate HOW OFTEN you use <u>performance-based tests</u> for the following purposes.

Purpose:	Never	Rarely	Sometimes	Often	Always
To inform clinical decisions	○	○	○	○	○
To evaluate patient progress	○	○	○	○	○
To communicate with patients	○	○	○	○	○
To justify service to payers	○	○	○	○	○
To communicate with other healthcare providers	○	○	○	○	○
For research purposes	○	○	○	○	○
To determine the effectiveness of a treatment/intervention	○	○	○	○	○
For practice management purposes (e.g., quality improvement)	○	○	○	○	○
For facility or provider accreditation	○	○	○	○	○

QUESTIONS ABOUT YOUR PERSPECTIVES ON USE OF OUTCOME MEASURES

Please rate the degree to which you agree with the following statements about <u>self-report surveys</u>.

Self-report surveys…	**Strongly disagree**	**Disagree**	**Neutral**	**Agree**	**Strongly Agree**
Are easy to make a part of my routine	○	○	○	○	○
Are a good use of my time	○	○	○	○	○
Are inexpensive	○	○	○	○	○
Can be quickly administered	○	○	○	○	○
Are helpful in acquiring insurance approvals	○	○	○	○	○
Can be administered with knowledge that I have	○	○	○	○	○
Are administered in a standardized way throughout my profession	○	○	○	○	○
Are easily scored and interpreted	○	○	○	○	○
Are easy to put into my medical record system	○	○	○	○	○
Are within my scope of practice	○	○	○	○	○
Are meaningful or motivating to my patients	○	○	○	○	○
Are a good use of my patients' time	○	○	○	○	○
Help me make clinical decisions	○	○	○	○	○
Help me communicate with other health care providers	○	○	○	○	○

Please rate the degree to which you agree with the following statements about <u>performance-based tests</u>.

Performance-based tests…	**Strongly disagree**	**Disagree**	**Neutral**	**Agree**	**Strongly Agree**
Are easy to make a part of my routine	○	○	○	○	○
Are a good use of my time	○	○	○	○	○
Are inexpensive	○	○	○	○	○
Can be quickly administered	○	○	○	○	○
Are helpful in acquiring insurance approvals	○	○	○	○	○
Can be administered using knowledge that I have	○	○	○	○	○
Are easy for me to conduct	○	○	○	○	○
Can be administered by me without additional help	○	○	○	○	○
Can be administered with equipment available in my clinic	○	○	○	○	○
Can be administered with space available in my clinic	○	○	○	○	○
Are administered in a standardized way throughout my profession	○	○	○	○	○
Are easily scored and interpreted	○	○	○	○	○
Are easy to put into my medical record system	○	○	○	○	○
Are within my scope of practice	○	○	○	○	○
Are meaningful or motivating to my patients	○	○	○	○	○
Are a good use of my patients' time	○	○	○	○	○
Help me make clinical decisions	○	○	○	○	○
Help me communicate with other health care providers	○	○	○	○	○

QUESTIONS ABOUT YOU

What is your highest education level completed? (choose one)

- ○ Bachelor's degree (PT)
- ○ Master's degree (MSPT)
- ○ Clinical doctorate (DPT)
- ○ PhD or other advanced degree

What year did you complete the National Physical Therapy Examination?

_______________(YYYY)

How long have you been practicing?

________________ years

Have you received any training specific to <u>outcomes measurement</u>? (check all that apply)

- ☐ Formal college or university coursework
- ☐ In-person continuing education courses
- ☐ Online continuing education courses
- ☐ Informal training from others
- ☐ Self-training
- ☐ Other: ______________
- ☐ None

What is your month and year of birth?

______________ Month ______________ Year

What is your gender? (check all that apply)

- ☐ Man
- ☐ Woman
- ☐ Gender diverse
- ☐ Non-binary
- ☐ Trans
- ☐ Prefer to self-describe: ___________________
- ☐ Prefer not to answer

What is your ethnicity? (choose one)

- ○ Hispanic or Latino
- ○ Not Hispanic or Latino
- ○ Prefer not to answer

What is your race? (check all that apply)

- ☐ American Indian or Alaskan Native
- ☐ Asian
- ☐ Black or African American
- ☐ Native Hawaiian or other Pacific Islander
- ☐ White
- ☐ Prefer to self-describe: ______________________
- ☐ Prefer not to answer

Thank you for taking this survey.
